# Chemistry of the Mouth

**Published:** 1873-05

**Authors:** M. McCarty


### CHEMISTRY OF THE MOUTH.

BY M. McCARTY.

*Read before the Tennessee Dental Association.*

In attempting to write an essay upon the Chemistry of the mouth I have purposely omitted the analysis of the teeth and saliva, for two obvious reasons. 1st, because I could not make it—hence it would not be original. 2nd, because you have all seen it time, and again and have it now in your text books ; consequently it would not be interesting to you.

I will therefore pass on, take up and endeavor to account for Dental Caries ; or more properly speaking the chemical corrosion of the teeth—for it is to chemical agents from without, that we must attribute the decay of the teeth.

It is to defective formation of the teeth in a great measure that we must look, in order to ascertain why it is that chemical agents act readily upon them.

Teeth that are covered with a dense and thick enamel, that s perfectly fused in the fissures during development resist all the ordinary corrodents for an indefinite period.

While those that are formed when there is not enough of the lime-salts elaborated to make the teeth of the proper density, and when the edges of the enamel do not fuse so as to perfectly close the fissures, or where there is a lack of vital energy to make and build up the structure perfectly, are subject to and will be frequently attacked by dental caries.

The first mentioned class of imperfect teeth are characterized by their whiteness and softness.

They are white and soft because there is a predominance of animal matter in them, and a consequent deficiency of earthy matter or lime-salts.

On account of the abundance of animal matter the atoms of earthy matter are ( comparatively speaking ) far apart; and are very liable to decay because the corrodents can easily pass in between the particles of lime salts.

Of the second imperfect class are those that have open fissures on the grinding Surface ; allowing chemical agents to pass into the dentine. We often meet with this class of teeth in young patients before decay sets in. A delicate thin bladed excavator readily passes in between the edges of the plates of the enamel; hence we can foretell the destruction of such teeth if the decay is not arrested. Query: is an operator justifiable in filling such teeth?

We now come to consider these "fell destroyers" these messengers of destruction — the agents that corrode and destroy these beautiful pearly gems of the mouth ; and cause them to stink worse than did the Augean Stable.

How are we to account for the presence of the agents of decay in the mouth. Acetic, Malic, Citric, Tartaric and Lactic acids are very abundant in the food we eat and drink. We are told however that none of these would do our teeth any serious injury in the diluted state we take them.

But we very well know that they set our teeth on edge, and what is that but the result of chemical corrosion? As the chemical union takes place between the acid and inorganic matter of the teeth the nerve fibrils are exposed ; then we have the oxygen of the air acting upon these bare tissues producing pain known as "teeth on edge."

We use acetic acid in one way or another almost daily. Vinegar is put in many things unnecessarily to sharpen our appetites and make us relish our food. But the great source whence comes its most injurious effect is the pickle.

The bottled pickle is most injurious in its effect. Pickles are generally made in brass kettles with acetic–acid vinegar, known as patent vinegar ; a part of the acid uniting with the copper forming acetate of copper. ( which is deadly poison.) This gives the pickles a beautiful green tint ; then they are prized by epicures for their sharp taste and fine color.

Young ladies are particularly fond of strong pickles, and eat them at improper times, just before retiring for instance, without taking anything in the mouth afterwards to neutralize or remove the acid ; the consequence is it unites with the lime of the teeth ; and it is for those who have been thus imprudent that we are called upon most frequently for our services. Malic acid is found in apples. Lactic acid in sour milk. Lemons, cranberries, gooseberries, strawberries, and raspberries, all owe the sourness to citric acid.

Tartaric acid exists in grapes. This is the acid that the fox objected to in the grapes that he could not get. It is much used in medicine to form acid refrigerant drinks and effervescing draughts. There are many other vegetable acids that are occasionally brought into contact with the teeth ; but we have said enough about the more important.

Next in order are the mineral acids that are taken through the mouth as medicines. And it is to these we must look for a great amount of the injury we are called upon to repair.

Physicians often find it neccessary to prescribe elixir vitriol, tincture of muriate of iron, and nitro muriatic acid, for diseases to which these medicines are severally applicable And generally be it said to their credit they warn their patients of the injurious effects of these agents upon their teeth, and give them instuuctions how to take them to prevent the injury. But alas ! how few patients follow strictly the directions given them.

During the progress of dyspepsia hydrochloric acid is frequently thrown into the mouth by eructations from the stomach. It being one of the constituents of the gastric juice.

In the course of many diseases there are elaborated into the system acids that find their way into the mouth by the salivary glands. For instance, in gout, rheumatism, ague, diabetes, gastro enteritis, the saliva contains lactic acid. And acetic acid in aphthæ, scrofula, scorbutus, smallpox, and indigestion. Uric acids in gouty affections. In fact during any depraved condition of the system acidity of the saliva is likely to occur.

